# HyMambaNet: Efficient Remote Sensing Water Extraction Method Combining State Space Modeling and Multi-Scale Features

**DOI:** 10.3390/s25247414

**Published:** 2025-12-05

**Authors:** Handan Liu, Guangyi Mu, Kai Li, Haowei Zhang, Yibo Sun, Hongqing Sun, Sijia Li

**Affiliations:** 1 Laboratory of Applied Disaster Prevention in Water Conservation Engineering of Jilin Province, Changchun Institute of Technology, Changchun 130103, China; liuhandan@stu.ccit.edu.cn (H.L.); sunyibo@stu.ccit.edu.cn (Y.S.); 20241371207@stu.ccit.edu.cn (H.S.); 2 Northeast Institute of Geography and Agroecology, Chinese Academy of Sciences, Changchun 130102, China; likai@stu.hrbnu.edu.cn (K.L.); lisj983@nenu.edu.cn (S.L.); 3 Department of Civil Engineering, The University of Hong Kong, Hong Kong 999077, China; u3011949@connect.hku.hk

**Keywords:** water body extraction, remote sensing, semantic segmentation, Mamba, HyMambaNet, state space model

## Abstract

Accurate segmentation of water bodies from high-resolution remote sensing imagery is crucial for water resource management and ecological monitoring. However, small and morphologically complex water bodies remain difficult to detect due to scale variations, blurred boundaries, and heterogeneous backgrounds. This study aims to develop a robust and scalable deep learning framework for high-precision water body extraction across diverse hydrological and ecological scenarios. To address these challenges, we propose HyMambaNet, a hybrid deep learning model that integrates convolutional local feature extraction with the Mamba state space model for efficient global context modeling. The network further incorporates multi-scale and frequency-domain enhancement as well as optimized skip connections to improve boundary precision and segmentation robustness. Experimental results demonstrate that HyMambaNet significantly outperforms existing CNN and Transformer-based methods. On the LoveHY dataset, it achieves 74.82% IoU and 88.87% F1-score, exceeding UNet by 7.49% IoU and 7.12% F1. On the LoveDA dataset, it attains 81.30% IoU and 89.99% F1-score, surpassing advanced models such as Deeplabv3+, AttenUNet, and TransUNet. These findings confirm that HyMambaNet provides an efficient and generalizable solution for large-scale water resource monitoring and ecological applications based on remote sensing imagery.

## 1. Introduction

With the intensifying impacts of global climate change and rapid urbanization, dynamic monitoring of water resources has become a pressing challenge for both ecosystem management and sustainable development [1,2]. Remote sensing, owing to its large spatial coverage and high temporal resolution, provides an effective means for capturing large-scale water body distribution and temporal variations [3,4,5]. However, small and morphologically complex water bodies—such as ponds, meandering rivers, and wetland patches—remain difficult to delineate precisely using traditional hydrological monitoring methods [6,7,8]. Accurate mapping of these small water bodies is critical for refined water resource management. It is also essential for understanding regional hydrological cycles, flood dynamics, and ecological restoration processes [9,10,11,12].

Early water body extraction approaches primarily relied on spectral indices, empirical thresholds, and shallow machine learning classifiers [13,14,15,16,17,18]. Although computationally efficient, these methods are highly sensitive to environmental noise, background heterogeneity, and manually defined parameters [19,20,21], which limits their applicability in complex natural and urban environments. With the development of deep learning, convolutional neural networks (CNNs) and end-to-end semantic segmentation frameworks have significantly advanced research on water body extraction [22,23,24,25,26,27]. Nevertheless, CNNs’ inherently local receptive fields hinder their ability to capture long-range spatial dependencies, often resulting in blurred boundaries and incomplete detection of small targets [28,29,30,31,32,33,34]. Transformer-based architectures partially address this issue by modeling global dependencies; however, their quadratic computational complexity restricts their application to high-resolution imagery [35,36,37]. Consequently, balancing global modeling capability with computational efficiency remains a major challenge.

Recently, state space models (SSMs), represented by Mamba, have demonstrated strong potential in visual sequence modeling due to their linear computational complexity and ability to capture long-range contextual dependencies efficiently [38,39,40,41,42,43,44]. These models alleviate the computational burden of Transformers while maintaining effective global context representation and boundary refinement. However, their application to high-resolution remote sensing image segmentation remains limited, particularly in scenarios with heterogeneous backgrounds and small-scale hydrological targets [45,46,47,48,49,50,51,52,53]. Although recent studies have introduced SSMs into various vision tasks, their integration with convolutional architectures for precise water body extraction is still relatively underexplored.

To address these challenges, this study proposes HyMambaNet, an efficient hybrid network that integrates state space modeling with multi-scale convolutional feature representation. Building upon the UNet architecture, the proposed method combines the global modeling capacity of Mamba-based modules with the local feature extraction strengths of CNNs. Additionally, the model incorporates multi-scale feature fusion, frequency-domain enhancement, and optimized skip connections. These additions improve segmentation robustness and boundary precision for complex water bodies in diverse environments. The remainder of this paper is organized as follows: Section 2 describes the datasets, overall framework, and methodological components of HyMambaNet. Section 3 presents the experimental setup and results. Section 4 provides a detailed discussion of the findings, and Section 5 concludes the paper.

## 2. Materials and Methods

### 2.1. Dataset

#### 2.1.1. LoveHY Dataset

**Figure 1:**
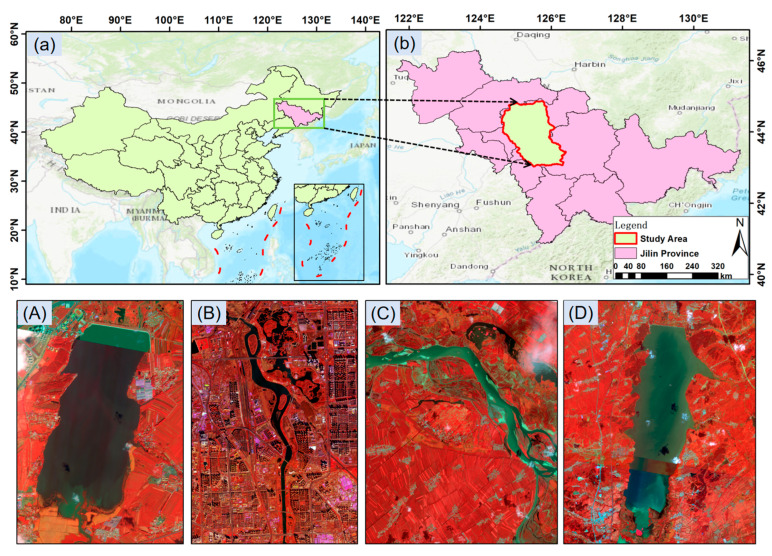
Location of the LoveHY dataset study area and examples of typical water body distribution in false-color composite images. (**a**) Administrative map of China showing the location of the study area; (**b**) Jilin Province with study area boundary; (**A**–**D**) False-color composite images of typical water bodies within the study area.

In this study, a high-resolution water body annotation dataset, LoveHY, was constructed using imagery from the Jilin-1 GF02 satellite. The dataset consists of 48 scenes covering Changchun City and its surrounding areas in Jilin Province (Figure 1). The images were acquired between June and October 2021, with a spatial resolution of 0.8 m. The study area is located in central Jilin Province, characterized mainly by plains and containing diverse representative water bodies such as rivers, lakes, and reservoirs. Figure 1 illustrates the location of the study area within China and Jilin Province, as well as the distribution of typical water bodies in false-color composite imagery.

**Figure 2:**
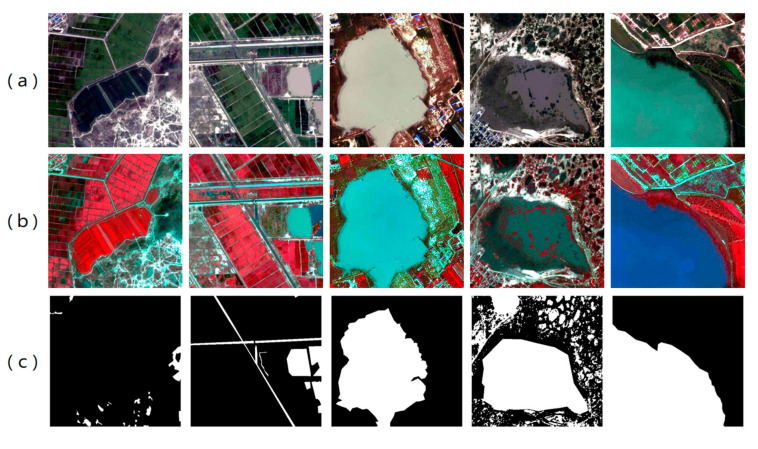
Examples from the LoveHY dataset. (**a**) Original true-color remote sensing images; (**b**) False-color composite images (NIR–R–G bands); (**c**) Corresponding manually annotated water body masks.

All images were subjected to rigorous preprocessing, including atmospheric correction, radiometric calibration, and geometric correction. Subsequently, fine-grained vector annotations of water bodies were generated by combining visual interpretation with ArcGIS 10.8 tools, resulting in high-quality ground truth labels. During sample construction, the original images were cropped into patches of 512 × 512 pixels, yielding a total of 5935 image–label pairs. To enhance the spectral contrast between water bodies and surrounding land cover, all cropped samples were represented using false-color composites of near-infrared, red, and green bands (Figure 2). Finally, the dataset was divided into training, validation, and test sets at a ratio of 70%, 15%, and 15%, respectively, ensuring both robust model training and reliable evaluation of generalization ability.

#### 2.1.2. LoveDA Dataset

**Figure 3:**
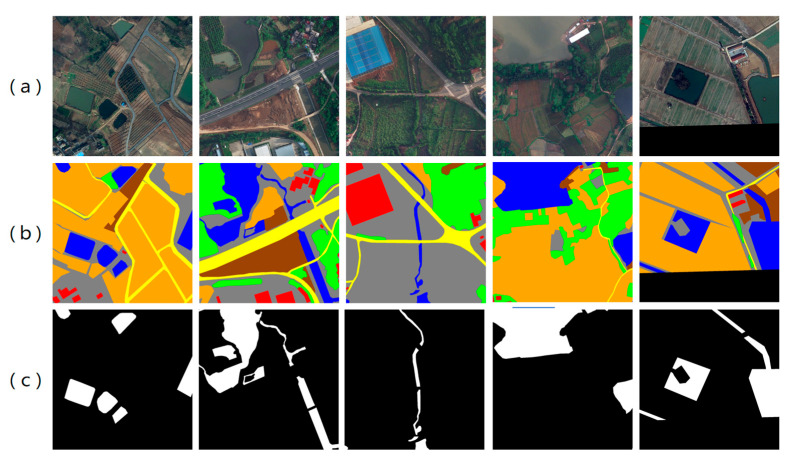
Examples from the LoveDA dataset. (**a**) Original true-color remote sensing images; (**b**) Visualization of seven-class annotations; (**c**) Binary water body mask annotations.

In addition, the publicly available LoveDA dataset [54] was employed in this study as a benchmark for comparative experiments. The dataset contains 5987 high-resolution remote sensing images collected from three Chinese cities—Nanjing, Changzhou, and Wuhan—with a spatial resolution of 0.3 m and an image size of 1024 × 1024 pixels. The original annotations include seven semantic categories: background, building, road, water, barren, forest, and agriculture (Figure 3). For the specific task of water body extraction, we adapted the labels by reassigning the water category to class 1 and merging all other categories into class 0 (background), thereby generating binary classification label maps suitable for our model.

To adapt the dataset for model input and reduce computational cost, all images were evenly cropped into four patches of 512 × 512 pixels. On this basis, a quality control process was performed to remove samples with evident noise, incomplete annotations, or those lacking valid water body information. After refinement, a total of 7990 image–label pairs were obtained. For fair and reproducible evaluation, the dataset was partitioned into training, validation, and test sets with a ratio of 70%, 15%, and 15%, respectively.

### 2.2. Overall Framework of HyMambaNet

**Figure 4:**
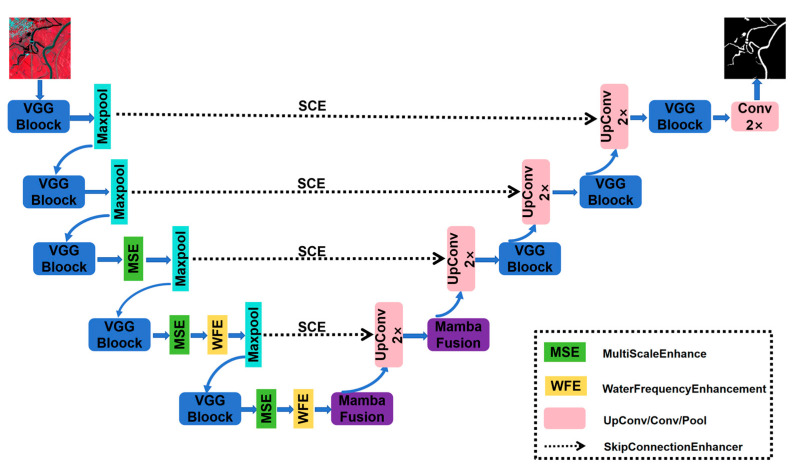
Overall architecture of the HyMambaNet model.

HyMambaNet is built upon the UNet architecture, integrating multiple innovative modules within a five-layer encoder–decoder structure to comprehensively enhance feature extraction and fusion capabilities (Figure 4). Specifically, multi-scale enhancement and frequency-domain enhancement modules are embedded in the encoder path to enrich feature representations; a state space model-based MambaFusion module is introduced at the bottleneck layer to capture long-range dependencies and model global context; and attention mechanisms are incorporated into the decoder path to optimize skip connections, thereby ensuring efficient fusion of shallow details and deep semantic information.

Water body extraction is particularly challenging due to its large-scale variations, blurred boundaries, and complex backgrounds. To address these issues, HyMambaNet has been carefully designed with targeted modules: the multi-scale enhancement module captures features under different receptive fields to handle scale disparities ranging from narrow streams to large lakes; the MambaFusion module, leveraging linear-complexity state space modeling, effectively addresses long-range dependencies in elongated water bodies such as rivers and water networks; the frequency-domain enhancement module preserves high-frequency information crucial for boundary recognition, alleviating detail loss caused by downsampling; and attention mechanisms are incorporated across multiple levels to enhance discrimination between water bodies and spectrally similar backgrounds such as shadows and dark vegetation. With these designs, HyMambaNet achieves superior accuracy and robustness in water body extraction under complex scenarios. The following sections will provide a detailed description of the structure and implementation of each key module.

### 2.3. Key Components of the Network

#### 2.3.1. Enhanced VGGBlock

**Figure 5:**
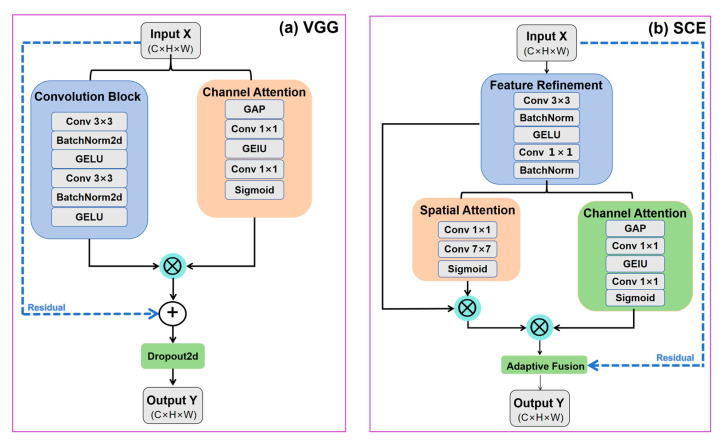
Core components of HyMambaNet. (**a**) Enhanced VGGBlock with channel attention and residual connections. (**b**) Skip Connection Enhancer (SCE) with dual attention mechanisms.

In HyMambaNet, an enhanced VGGBlock is adopted as the basic feature extraction unit. Although the original VGG architecture is effective in hierarchical feature learning, its direct application to water body segmentation often suffers from gradient vanishing and insufficient feature discrimination in complex environments. To address these issues, we introduce an enhanced VGGBlock (Figure 5), which integrates improved convolutional layers, residual learning, and lightweight channel attention.

The module employs two sequential 3 × 3 convolutional layers, each followed by batch normalization (BN) and GELU activation, to stabilize training and provide smoother gradients. This improves the model’s ability to capture fine water boundaries and reduces over-smoothing effects.

The two convolutional operations are defined as:

(1) $$F_{1}=\mathrm{GELU}(\mathrm{BN}({\mathrm{Conv}}_{3\times 3}(x)))$$

(2) $$F_{2}=\mathrm{GELU}(\mathrm{BN}({\mathrm{Conv}}_{3\times 3}(F_{1})))$$

where

- $x\in {\mathbb{R}}^{C_{\mathrm{in}}\times H\times W}$ is the input feature map, with $C_{\mathrm{in}}$ input channels and spatial size H × W.
- ${\mathrm{Conv}}_{3\times 3}$ (·) denotes a 3 × 3 convolution with padding = 1 to preserve spatial resolution.
- BN(·) denotes batch normalization.
- GELU(·) denotes the Gaussian Error Linear Unit activation.
- F_1_ and F_2_ are intermediate feature maps with $C_{\mathrm{out}}$ channels.

To enhance channel-wise feature discrimination, a lightweight channel attention mechanism is integrated. It aggregates global spatial information through global average pooling and models inter-channel dependencies using a bottleneck structure with a reduction ratio r = 8. This emphasizes water-related spectral responses and suppresses background interference.

A residual connection is employed to fuse the enhanced features with the input. When the input and output channels differ, a 1 × 1 convolution is used to align channel dimensions. Spatial dropout (*p* = 0.1) is applied before the residual addition to alleviate overfitting. The final output is computed as:

(3) $$y=\mathrm{Dropout}(F_{2^{\prime }})+F_{\mathrm{match}}(x)$$

where

- $F_{\mathrm{match}}(x)=\left\{\begin{array}{ll} x, & C_{\mathrm{in}}{=C}_{\mathrm{out}}\\ {\;\mathrm{Conv}}_{1\times 1}(x), & C_{\mathrm{in}}\neq {\;C}_{\mathrm{out}} \end{array}\right.$
- Dropout(F_2_) refers to spatial dropout applied to the enhanced feature map.
- $F_{\mathrm{match}}(x)\;$ ensures that the residual branch aligns with the enhanced branch in channel dimension.
- *y* is the final output of the enhanced VGGBlock.

This enhanced VGGBlock provides an effective receptive field equivalent to 5 × 5, enabling the extraction of multi-scale boundary cues. The integration of channel attention improves adaptive feature selection, while the residual structure facilitates stable optimization and preserves fine-grained details critical for accurate water body delineation.

#### 2.3.2. Skip Connection Enhancer (SCE)

In UNet-like architectures, skip connections play a critical role in transferring spatial details from the encoder to the decoder. However, directly concatenating shallow encoder features with deeper decoder representations often introduces a semantic gap. Shallow features primarily encode local textures and boundary information, whereas deeper features emphasize high-level semantics. This mismatch may cause noise amplification, blurred boundaries, and the loss of small water bodies—issues that are particularly evident in remote sensing water extraction.

To address this problem, we propose the SCE module (Figure 5), which refines encoder features before fusion by integrating lightweight convolutional refinement, spatial attention, and adaptive residual fusion.

The SCE module first applies depthwise separable convolutions to obtain a refined feature representation $F_{\mathrm{refine}}$ with reduced computational cost. To better emphasize water-related boundary cues, a spatial attention mechanism with an extended receptive field is introduced:

(4) $$M_{\mathrm{spatial}}=\sigma \left({\mathrm{Conv}}_{7\times 7}\left({\mathrm{Conv}}_{1\times 1}(F_{\mathrm{refine}})\right)\right)$$

where

- $F_{\mathrm{refine}}$ is the refined encoder feature map.
- ${\mathrm{Conv}}_{1\times 1}$ (·) denotes a 1 × 1 convolution for channel reduction and feature mixing.
- ${\mathrm{Conv}}_{7\times 7}$ (·) denotes a large-kernel convolution for capturing long-range spatial context.
- σ (·) is the sigmoid activation.
- $M_{\mathrm{spatial}}$ is the resulting spatial attention map.

The attended feature is further enhanced using channel attention to emphasize channels related to water surfaces while suppressing responses from spectrally similar backgrounds. The resulting feature is denoted $F_{\mathrm{att}}$. An adaptive skip fusion is then introduced to balance the detailed spatial information from the encoder and the enhanced semantic cues:

(5) $$Y=\alpha \cdot F_{\mathrm{att}}+(1-\alpha )\cdot X_{\mathrm{skip}}$$

where

- $F_{\mathrm{att}}$ is the attention-enhanced feature after spatial and channel attention.
- $X_{\mathrm{skip}}$ is the original skip-connection feature.
- α∈[0, 1] is a learnable weight initialized to 0.5.
- Y is the output feature of the SCE module.

This adaptive formulation enables the network to dynamically adjust the contribution of spatial details and semantic enhancement based on feature characteristics. By refining encoder features prior to fusion, the SCE module effectively reduces the semantic gap, suppresses background noise, reinforces water boundaries, and stabilizes training during decoding.

#### 2.3.3. MambaFusion

**Figure 6:**
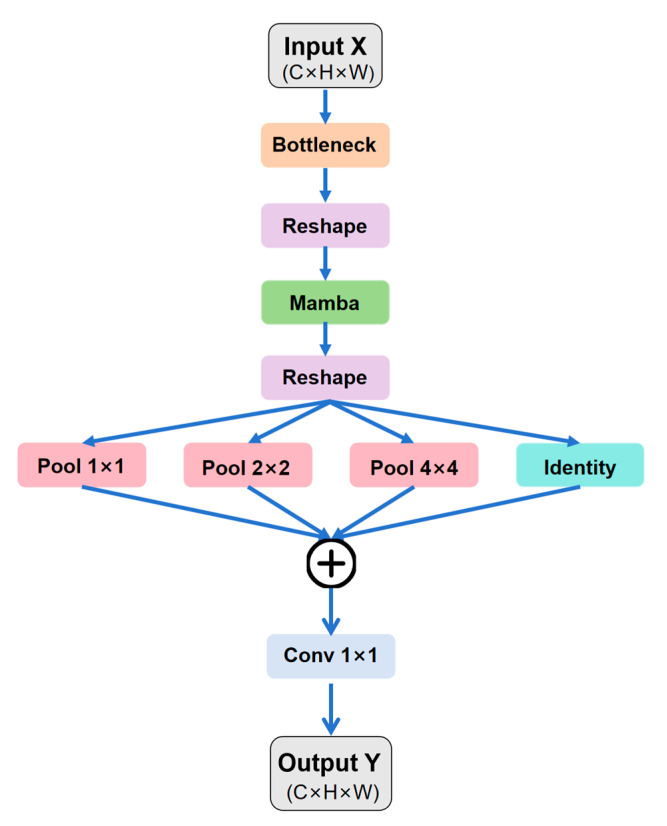
Architecture of the MambaFusion module in HyMambaNet. The module integrates bottleneck projection, Mamba-based sequence modeling, and multi-scale spatial pooling to enhance feature fusion.

Due to the significant spatial and morphological variability of water bodies from interconnected large lakes to narrow, winding river networks traditional CNNs are limited by their local receptive fields and struggle to capture long-range dependencies effectively. Although Transformer architectures possess strong global modeling capabilities, their quadratic computational complexity O(N^2^) incurs excessive overhead when processing high-resolution remote sensing images, limiting large-scale applications. To address this issue, we propose the MambaFusion module (Figure 6), which leverages Mamba’s linear-complexity sequence modeling to efficiently aggregate global context and fuse features in the deeper layers of the decoder.

MambaFusion is constructed based on the SSM, whose discrete form can be expressed as:

(6) $$h_{t}=Ah_{t-1}+Bx_{t}$$

(7) $$y_{t}=Ch_{t}$$

where

- $h_{t}$ denotes the hidden state.
- $x_{t}$ and $y_{t}$ represent the input and output sequences.
- *A*, *B*, and *C* are the system matrices. Unlike conventional SSMs with fixed parameters, Mamba dynamically adapts these matrices using a selective scanning mechanism (S6), enabling context-aware parameter evolution based on input features.

From a structural perspective, MambaFusion combines the global modeling capability of the Mamba branch with the local texture representation learned by the multi-scale pooling branch, while simultaneously preserving fine spatial details through a learnable residual pathway. The final fused feature is computed as:

(8) $$F_{\mathrm{out}}=F_{\mathrm{mamba}}+F_{\mathrm{pool}}+\lambda F_{\mathrm{res}}$$

where

- $F_{\mathrm{mamba}}$ is the global-context feature modeled by the Mamba branch.
- $F_{\mathrm{pool}}$ is the multi-scale pooled local feature.
- $F_{\mathrm{res}}$ is the residual feature ensuring boundary preservation.
- λ is a learnable scalar balancing global and local contributions.

This fusion strategy effectively combines long-range structural information with high-resolution spatial details, enabling robust water body decoding even under complex morphological variations.

#### 2.3.4. Multi-Scale Enhancement (MSE)

**Figure 7:**
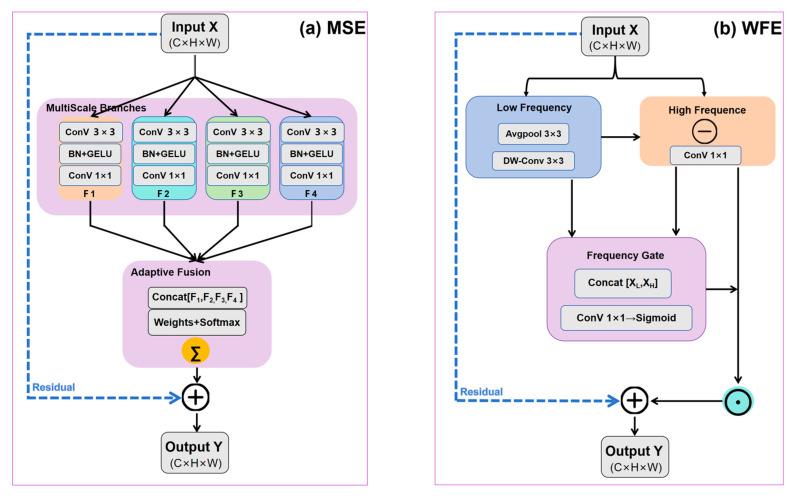
Dedicated enhancement modules for water body extraction in HyMambaNet. (**a**) Multi-Scale Enhancement (MSE) for capturing multi-scale features. (**b**) Water Frequency Enhancement (WFE) for enhancing frequency-domain water features.

Water bodies in remote sensing images exhibit significant scale variation, ranging from narrow streams of only a few pixels to large lakes spanning hundreds of pixels. Traditional CNNs, with fixed receptive fields, struggle to simultaneously capture local details and global context, which can adversely affect segmentation accuracy. To address this issue, we propose the MSE module (Figure 7), which efficiently captures features at different scales using parallel dilated convolutions.

Specifically, given an input feature map $F_{i}$, the MSE module employs D parallel branches, each utilizing dilated convolutions to obtain receptive fields at different scales:

(9) $$F_{i}={\mathrm{Conv}}_{3\times 3}^{d_{i}}(F),\;\;\;i\in \{0,1,\ldots ,D-1\}$$

where

- *F* is the input feature map.
- ${\mathrm{Conv}}_{3\times 3}^{d_{i}}$ (⋅) denotes a 3 × 3 dilated convolution with dilation rate.
- D is the number of dilation branches.
- F is the feature extracted at scale.

These parallel branches produce multi-scale representations covering receptive fields from fine local details (3 × 3) to broader region-level context (e.g., 17 × 17). To adaptively integrate information from different scales, an attention-based fusion mechanism is introduced. A learnable weight vector 𝑤 is used to compute normalized fusion weights:

(10) $$F_{\mathrm{out}}=\sum_{i=0}^{D-1}\frac{e^{w_{i}}}{\sum_{j=0}^{D-1}e^{w_{j}}}F_{i}+F$$

where

- $w_{i}$ is the learnable weight for scale.
- The term is $\frac{e^{w_{i}}}{\sum_{j}e^{w_{j}}}$ a softmax ensuring normalized scale weights.
- F provides a residual connection for stability.
- $F_{\mathrm{out}}$ is the fused multi-scale feature.

This adaptive weighting enables the module to selectively emphasize the most informative receptive fields for water body segmentation, improving robustness to varying object sizes and shapes.

#### 2.3.5. Water Frequency Enhancement (WFE)

Accurate water boundary identification is a critical challenge in remote sensing image segmentation, especially in complex backgrounds such as wetlands and seasonal rivers. Traditional CNNs tend to lose high-frequency information during successive downsampling, resulting in blurred boundaries. To address this issue, we propose the WFE module (Figure 7), which is designed based on the frequency-domain characteristics of water bodies: the interior of water regions is dominated by low-frequency components, whereas boundaries and fine structures are rich in high-frequency features.

Given an input feature map *F*, the WFE module first decomposes it into low- and high-frequency components:

(11) $$F_{\mathrm{low}}=\mathrm{DWConv}(F)$$

(12) $$F_{\mathrm{high}}={\mathrm{Conv}}_{1\times 1}(F)$$

where

- DWConv(·) denotes depthwise convolution capturing low-frequency components.
- ${\mathrm{Conv}}_{1\times 1}(\cdot )$ extracts fine-grained high-frequency details.
- $F_{\mathrm{low}}$ and $F_{\mathrm{high}}$ represent low- and high-frequency features, respectively.

To selectively enhance features related to water boundaries while suppressing noisy responses, we introduce an adaptive gating mechanism. The low- and high-frequency features are concatenated and passed through a convolution followed by a sigmoid activation to generate a gating mask:

(13) $$F_{\mathrm{out}}=F_{\mathrm{low}}+\left(\sigma \left(\mathrm{Conv}(\mathrm{Concat}[F_{\mathrm{low}},F_{\mathrm{high}}])\right)⊙F_{\mathrm{high}}\right)$$

where

- Concat[·] denotes channel-wise concatenation.
- Conv(·) is a convolution that produces the gating feature.
- σ(·) is the sigmoid activation controlling enhancement strength.
- ⊙ denotes element-wise multiplication.
- $F_{\mathrm{out}}$ is the frequency-enhanced output feature.

This adaptive mechanism dynamically amplifies high-frequency boundary cues while retaining structural consistency in low-frequency regions. As a result, the WFE module enhances fine water boundaries and improves segmentation accuracy in complex background environments.

### 2.4. Experimental Setup

#### 2.4.1. Water Body Extraction Workflow

**Figure 8:**
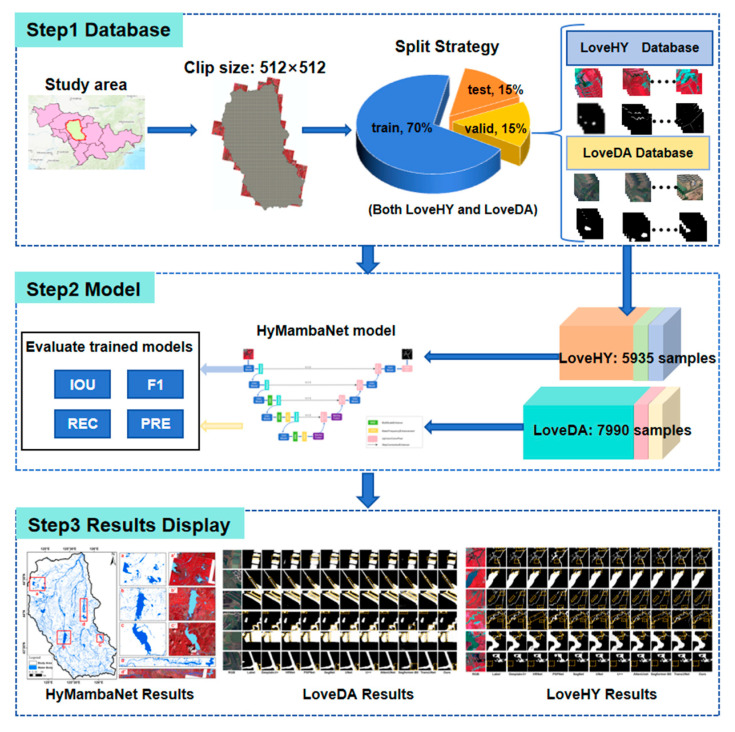
Workflow of water body extraction from multi-source remote sensing datasets.

In this study, we employed both the private LoveHY dataset and the public LoveDA dataset to train and evaluate HyMambaNet, aiming to verify its scalability and robustness. To provide a clearer illustration of the implementation process, Figure 8 presents the complete workflow for water body extraction. This workflow is structured into three operational stages: dataset integration and preprocessing, deep learning model training, and segmentation result generation. By following this standardized procedure, an automated pipeline from raw remote sensing imagery to water body extraction results is realized.

#### 2.4.2. Experimental Environment and Parameter Settings

All experiments were conducted on a high-performance server equipped with an Intel(R) Xeon(R) Gold 6430 16-core CPU (Intel, Santa Clara, CA, USA) and an NVIDIA RTX 4090 GPU (NVIDIA, Santa Clara, CA, USA). The software environment consisted of Python 3.10 and PyTorch 2.2.1, along with CUDA 11.8 and cuDNN 8.9.5, ensuring efficient acceleration for both training and inference.

During training, Binary Cross-Entropy (BCE) loss was adopted for optimizing the binary segmentation task of distinguishing water from non-water regions. All models were trained for 200 epochs with an Adam optimizer, using a batch size of 8 and an initial learning rate of 0.0001. The learning rate was dynamically adjusted using a cosine-annealing schedule to improve convergence stability. The input images were resized to 512 × 512, normalized to [0, 1], and augmented with random flipping, rotation, and color jitter to increase robustness.

To ensure fairness, all comparison methods were trained using the same training, validation, and test split, the same data augmentation strategy, and identical hyperparameter settings unless otherwise specified. The evaluation metrics used in this study are presented in Section 2.5.

#### 2.4.3. Computational Complexity and Efficiency Analysis

To provide a rigorous justification of the computational advantages introduced by the proposed MambaFusion module, we analyze both the theoretical complexity and the empirical efficiency compared with a Transformer-based fusion module.

(1) Theoretical Complexity Analysis

Transformer self-attention requires computing pairwise interactions among all spatial to kens, resulting in a quadratic computational complexity:

$$O(N^{2}),$$

where N = H × W denotes the number of spatial positions in the feature map. Such quadratic growth becomes computationally prohibitive when processing high-resolution remote sensing images, which often exceed millions of pixels.

In contrast, the Mamba state-space model (SSM) employed in MambaFusion updates tokens through a selective scanning mechanism, where each token interacts only with a small number of learned state parameters. This leads to a linear complexity:

O(N),

meaning computation grows proportionally with image size. This linear formulation is significantly more efficient and scalable for high-resolution water body segmentation tasks.

(2) Empirical Efficiency Comparison

To validate the theoretical advantage in practice, we further compare the computational cost of a standard Transformer-based fusion module and the proposed MambaFusion under identical settings. The results are summarized in Table 1.

### 2.5. Model Performance Evaluation Metrics

To comprehensively assess the performance of HyMambaNet in image segmentation tasks, four evaluation metrics were employed: precision, recall, IoU, and F1-score. The corresponding calculation formulas are as follows.

(14) $$\mathrm{Precision}=\frac{\mathrm{TP}}{\mathrm{TP}+\mathrm{FP}}$$

(15) $$\mathrm{Recall}=\frac{\mathrm{TP}}{\mathrm{TP}+\mathrm{FN}}\;$$

(16) $$\mathrm{IoU}=\frac{\mathrm{TP}}{\mathrm{TP}+\mathrm{FP}+\mathrm{FN}}\;$$

(17) $$F1=2\times \frac{\mathrm{Precision}\times \mathrm{Recall}}{\mathrm{Precision}+\mathrm{Recall}}$$

Here, TP (True Positive) refers to positive samples correctly predicted as positive, FP (False Positive) refers to negative samples incorrectly predicted as positive, TN (True Negative) refers to negative samples correctly predicted as negative, and FN (False Negative) refers to positive samples incorrectly predicted as negative.

## 3. Results

### 3.1. Visualization of Segmentation Results for Water Bodies with Different Scales and Morphologies

**Figure 9:**
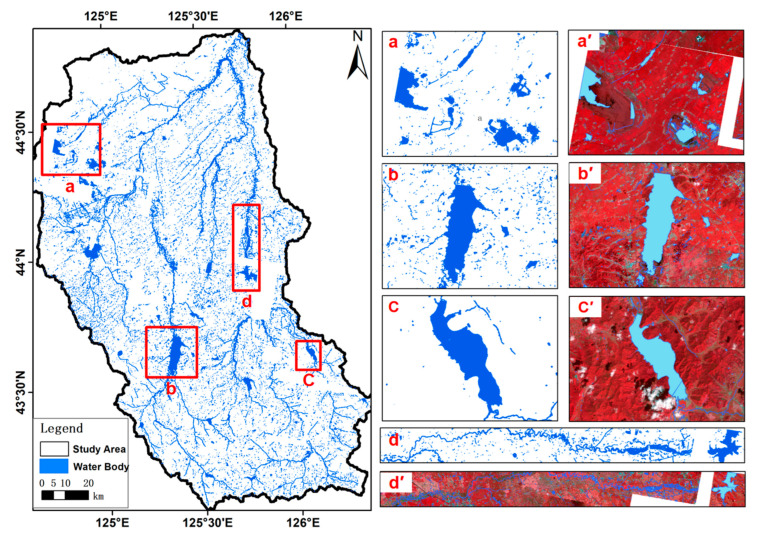
Water body extraction results of HyMambaNet on the LoveHY dataset. The main map (left) shows the full water body distribution in the study area. The right panel shows zoomed-in examples (**a**–**d**), comparing the model’s prediction map (**a**–**d**) to the corresponding water body label map (**a′**–**d′**).

Figure 9 Representative segmentation results of HyMambaNet on the self-constructed LoveHY dataset. The right panel corresponds to four representative types of water body subregions: (a) small, dispersed water bodies; (b) medium-sized lakes with regular boundaries; (c) morphologically complex water bodies; and (d) elongated rivers. For each subregion, the model’s prediction map (a–d) and the corresponding water body label map (a′–d′) are provided to visually highlight the spectral differences and spatial characteristics of different water body types.

The left panel shows the complete water body distribution map obtained by stitching and merging predictions from multiple subregions, where blue pixels indicate the extracted water bodies, including lakes, reservoirs, rivers, and ponds. It can be observed that HyMambaNet accurately captures water targets of varying scales and morphologies, achieving high precision from large lakes to narrow rivers. These results validate the model’s effectiveness and robustness in multi-scale feature learning and spatial generalization.

### 3.2. Ablation Study

To comprehensively evaluate the effectiveness of each module in HyMambaNet, a systematic ablation study was conducted. The experimental settings were kept consistent with the main comparative experiments, including dataset partitioning and training configurations. The approach used the classical UNet as the baseline model (A0), and modules were gradually introduced to assess their contribution to overall performance. Table 2 summarizes the results for different module combinations.

The results indicate that each module positively contributed to performance improvements. Introducing the VGGBlock (A1) increased the IoU from 67.33% to 68.92%, with a corresponding improvement in F1-score, demonstrating that deep feature extraction enhances boundary localization. The MSE module (A2) further improved recall and IoU (70.15%), validating its effectiveness in handling scale variations. The Skip Connection Enhancement module (A3) significantly optimized feature fusion, boosting IoU to 71.84%. The WFE module (A4) strengthened boundary texture features, resulting in a 1.45% IoU increase. The addition of the MambaFusion module (A5) effectively modeled global context, raising IoU to 74.18%. Finally, the complete model integrating all components (A6) achieved an IoU of 74.82% and an F1-score of 88.87% on the LoveHY dataset, representing improvements of 7.49% and 7.12% over the baseline U-Net, respectively. Overall, the Water Frequency Enhancement module and the SCE contributed most significantly, while the MambaFusion module played a key role in coordinating global modeling across components.

**Figure 10:**
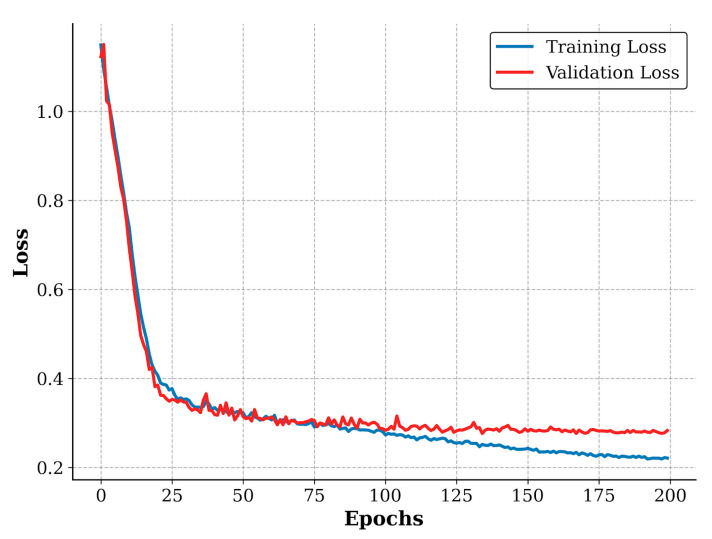
Visualization of the training process. The convergence curves of training loss and validation loss on the LoveHY dataset demonstrate the stability of the training phase.

To further verify the stability of the training process, we plotted the loss convergence curves of the proposed HyMambaNet on the LoveHY dataset, as shown in Figure 10. It can be observed that both training and validation losses decrease steadily and converge effectively as the epochs increase. Notably, the validation loss follows the training loss closely without exhibiting significant fluctuations or divergence, indicating that the model training is stable and free from severe overfitting.

### 3.3. Comparative Study on the LoveHY Dataset

To evaluate the effectiveness of HyMambaNet in water body segmentation, we conducted a systematic comparison with several classical and state-of-the-art semantic segmentation methods, including Deeplabv3+ [55], HRNet [32], PSPNet [56], SegNet [57], UNet [25], U++ [27], AttenUNet [58], SegFormer-B0 [59], and TransUNet [28]. All methods were trained under identical hyperparameters, data augmentation strategies, and loss functions to ensure a fair comparison. Table 3 summarizes the quantitative evaluation results of these methods on the LoveHY dataset.

As shown in the results, HyMambaNet achieves the best performance across all evaluation metrics. Specifically, our method attains a precision of 86.88%, significantly higher than the other approaches, indicating stronger reliability in identifying water pixels. The recall reaches 90.97%, demonstrating the model’s ability to effectively capture a larger number of true water targets. IoU, as a core metric for segmentation tasks, reaches 74.82% with HyMambaNet, representing a 3.37% improvement over the second-best method, TransUNet (71.45%), further validating the high consistency between predictions and ground truth. Additionally, our method achieves an F1-score of 88.87%, achieving a superior balance between precision and recall. In contrast, methods such as PSPNet and SegFormer-B0 perform poorly in complex water body scenarios, as evidenced by their lower IoU and F1 scores.

**Figure 11:**
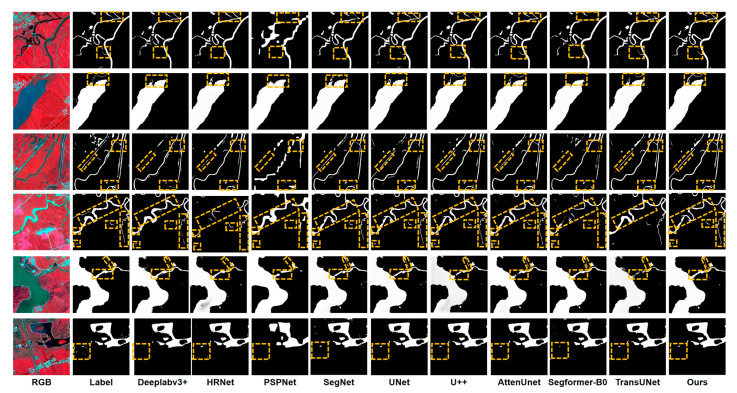
Visualization of segmentation results by different methods on the LoveHY dataset.

To further visually validate model performance, Figure 11 presents the segmentation results of all methods. It can be observed that traditional CNN-based methods (e.g., PSPNet and SegNet) often miss or misclassify narrow rivers or shadowed regions. Although Deeplabv3+ and HRNet show improvements in overall performance, they still produce fragmented predictions in complex river network scenes. UNet and its variant U++ provide better contextual modeling but remain slightly insufficient in boundary details. AttenUNet and SegFormer-B0 demonstrate certain advantages but still exhibit noticeable limitations on the LoveHY dataset. In comparison, HyMambaNet generates clearer, more continuous segmentation results that closely match the ground truth across different types of water bodies, including large lakes and intricate river networks, fully demonstrating its robustness and superiority in complex remote sensing scenarios.

### 3.4. Comparative Study on the LoveDA Dataset

To further evaluate the generalization capability and effectiveness of HyMambaNet, we conducted rigorous experiments on the public LoveDA dataset and compared it with several mainstream semantic segmentation methods, including Deeplabv3+, HRNet, PSPNet, SegNet, UNet, U++, AttenUNet, SegFormer-B0, and TransUNet. All models were trained under identical hyperparameters, data augmentation strategies, and loss functions to ensure a fair comparison. Table 4 summarizes the quantitative comparison results of these methods on the LoveDA dataset.

The results indicate that HyMambaNet achieves the best performance across all key metrics. Specifically, it attains a precision of 88.29%, an IoU of 81.30%, and an F1-score of 89.99%, significantly outperforming other methods. Meanwhile, the recall reaches 91.79%, slightly higher than AttenUNet (91.51%) and Deeplabv3+ (90.21%). These results demonstrate that HyMambaNet can maintain high recognition accuracy while effectively reducing missed detections, enabling more comprehensive identification of water pixels. Overall, the method achieves state-of-the-art performance on the LoveDA dataset, particularly excelling in balancing precision and recall, thereby obtaining the highest IoU and F1-score.

**Figure 12:**
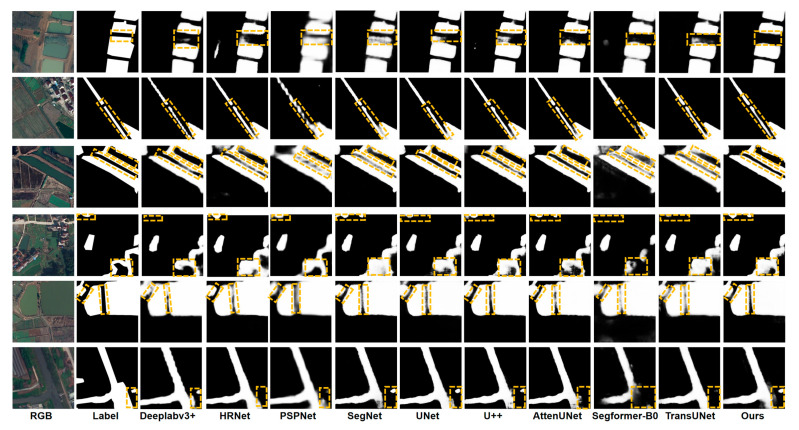
Visualization of segmentation results by different methods on the LoveDA dataset.

To visually illustrate segmentation performance, Figure 12 presents the results of different methods on the LoveDA dataset. It can be observed that traditional CNN-based methods (e.g., PSPNet and SegNet) often suffer from missed detections and misclassifications in complex backgrounds. Although Deeplabv3+ and HRNet improve overall performance, they still struggle with fine-grained boundary delineation and the identification of small-scale water bodies. U-Net and U++ perform well in contextual modeling but remain less precise in boundary localization. AttenUNet and TransUNet demonstrate strong performance in certain scenarios, yet their overall stability is limited, while SegFormer-B0 shows limitations in learning water body features. In contrast, HyMambaNet consistently generates segmentation masks that closely match the ground truth, producing clearer, more continuous boundaries with minimal misclassification across both large lakes and intricate river networks. These visual results further corroborate the quantitative advantages, fully demonstrating HyMambaNet’s superior robustness and generalization capability in complex public remote sensing scenarios.

### 3.5. Failure Cases and Error Analysis

**Figure 13:**
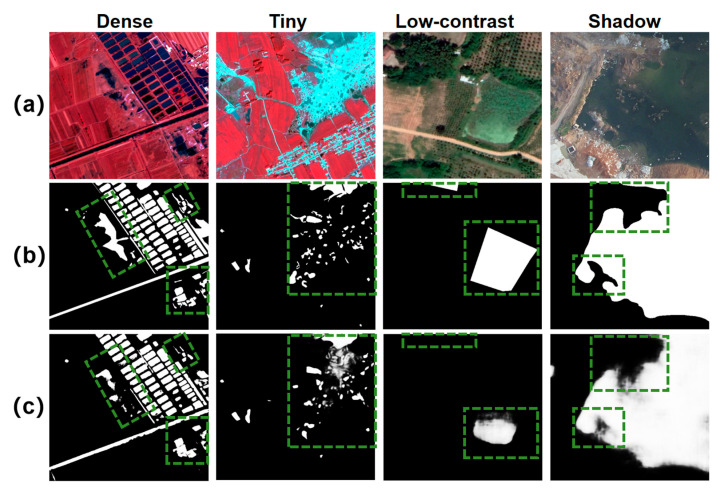
Visualization of typical failure cases. (**a**) Original images; (**b**) Ground truth labels; (**c**) HyMambaNet predictions. Columns represent four challenging scenarios: dense adjacent objects, tiny scattered targets, low-contrast water bodies, and shadow interference. Green dashed boxes highlight the specific regions of incorrect predictions.

Although HyMambaNet achieves state-of-the-art performance on both LoveHY and LoveDA datasets, several challenging scenarios still lead to incorrect predictions, as summarized in Figure 13. Specifically, in scenarios with dense aquaculture ponds (Column 1), the model struggles to separate closely adjacent water bodies, occasionally leading to boundary adhesion or the omission of narrow dividers due to limited spatial resolution. Similarly, when water bodies are extremely small and scattered (Column 2), or exhibit low contrast with surrounding vegetation (Column 3), the model fails to capture the complete water features, resulting in missed detections (False Negatives). Conversely, a typical False Positive case is observed in regions with strong shadow interference (Column 4). In this case, large shadows cast by terrain or mining pits share similar color and intensity characteristics with deep water, causing the model to incorrectly classify these non-water regions as water. These failure cases demonstrate that extreme small-scale targets, low-contrast water–land transitions, and highly heterogeneous backgrounds (especially shadows) remain challenging for HyMambaNet. Future improvements may focus on multi-source fusion (e.g., SAR + optical), boundary-aware loss functions, and targeted data augmentation to further enhance robustness in these complex conditions.

## 4. Discussion

### 4.1. Contribution of Key HyMambaNet Modules to Water Body Segmentation Performance

The ablation study quantitatively evaluates the contribution of each core module in HyMambaNet. The UNet baseline, denoted as Model A0, achieves an IoU of 67.33%. After introducing the enhanced VGGBlock (Model A1), the IoU increases to 68.92%, corresponding to an improvement of 1.59%. Incorporating the MSE module (Model A2) further raises the IoU to 70.15%, which is 2.82% higher than the baseline. With the subsequent integration of SCE (Model A3) and WFE (Model A4), the IoU increases to 71.84% and 73.29%, respectively. Finally, the complete architecture with the MambaFusion module (Model A5) yields an overall IoU gain of approximately 7% relative to the baseline (Table 2). This stepwise performance improvement indicates that each module contributes a tangible benefit rather than serving as a purely conceptual extension.

Regarding local feature modeling, our MSE and WFE modules play roles similar to the multi-scale aggregation components reported in recent studies [21,22,26,31,60,61]. For example, in the Multi-Scale Feature Aggregation Network proposed by Hu et al., the progressive introduction of fusion modules increases the mIoU on river segmentation tasks from 90.94% with a ResNet-50 baseline to 95.94%, corresponding to an improvement of 5.00% [60]. Similarly, SPFDNet improves the IoU on the GF2020 dataset from 74.78% with a UNet baseline to 78.93%, yielding a gain of 4.15% through spatial partitioning [61]. Consistent with these findings, the combination of MSE and WFE in our model results in a cumulative IoU gain of nearly 6 percentage points over the baseline. This level of improvement is comparable to that of state-of-the-art frameworks and highlights the effectiveness of our boundary-aware design for complex water bodies.

With respect to global context modeling, the MambaFusion module introduces a state-space mechanism that captures long-range dependencies with linear computational complexity. In our ablation study, adding MambaFusion to the locally enhanced backbone (from Model A4 to A5) provides an additional IoU gain of roughly 1–2%, indicating that global context contributes non-trivially beyond local enhancements alone. This observation is consistent with trends reported in other Mamba-based dense prediction models. For example, RS-Mamba attains an F1-score of 91.10% and an IoU of 83.66% on the LEVIR-CD dataset, surpassing Transformer-based methods such as ChangeFormer, whose F1-score and IoU reach 90.40% and 82.47%, respectively, under comparable parameter budgets [52]. In the broader computer vision field, Vision Mamba (Vim) has been shown to run about 2.8 times faster than DeiT while reducing GPU memory consumption by approximately 86.8% on high-resolution benchmarks [42]. Taken together, these results support the view that state-space models offer a more favorable trade-off between accuracy and computational cost than conventional CNNs [23,25,32] or Transformers with quadratic complexity [34,39,59]. Within this context, the performance gains observed in HyMambaNet are consistent with the growing body of evidence in favor of Mamba-based architectures for high-resolution remote sensing.

In summary, the design strength of HyMambaNet lies not only in the performance enhancement of individual modules but also in the organic integration of local feature refinement (VGGBlock, MSE, SCE, WFE) and efficient global context modeling (MambaFusion). Unlike existing CNN-based networks that focus primarily on deep feature extraction [21,22,24,26,31] or Transformer-based architectures that emphasize heavy global attention [34,39,59], HyMambaNet provides a synergistic hybrid solution. This approach secures a sizable accuracy lead over the baseline, enabling robust segmentation of complex backgrounds and multi-scale water targets with clear advantages in both accuracy and robustness.

### 4.2. Performance and Generalization Analysis Across Different Datasets

Results from the LoveHY and LoveDA benchmarks strongly support HyMambaNet’s stability and generalization potential. The LoveHY dataset presents a challenging mix of high-resolution scenes, ranging from small ponds and narrow rivers to large, spatially complex lakes. Here, HyMambaNet demonstrates a distinct advantage in IoU and F1 scores, particularly when isolating small water bodies or tracing irregular shorelines. While classical convolutional networks such as SegNet and PSPNet frequently overlook narrow rivers or struggle in shadowed regions, Transformer-based counterparts like SegFormer-B0 and TransUNet often compromise boundary sharpness despite their global context awareness. By integrating frequency-domain enhancement with refined skip connections, our model delivers more complete segmentation maps with crisper land–water interfaces—accuracy that is vital for shoreline delineation and the restoration of small water bodies [2,8,9,12].

Further evaluation on the LoveDA dataset assessed the model’s adaptability across diverse urban landscapes characterized by highly heterogeneous land cover. Even in these complex settings, HyMambaNet maintained the most effective balance between precision, IoU, and F1-score, proving its ability to handle varying sensor types and resolutions. Conversely, DeepLabV3+ often sacrificed precision for higher recall, while HRNet-variants maintained precision but suffered from significant omission errors in cluttered backgrounds. Such errors are not merely statistical; they can introduce dangerous biases into flood risk mapping or urban surface water inventories [10,11]. Collectively, this consistent performance across datasets highlights HyMambaNet’s utility for fine-grained monitoring. It provides a robust foundation for practical applications ranging from lake storage estimation to urban flood analysis [2,9,11,12], ensuring reliable operation in heterogeneous environments.

### 4.3. Limitations and Future Work

Although HyMambaNet performs well across multiple experiments, it still has several limitations. The introduction of multiple enhancement modules improves boundary delineation and multi-scale feature modeling, but it also increases computational load and memory usage. This may create efficiency bottlenecks for large-area remote sensing applications and near-real-time monitoring. From a water resources management perspective, this issue is particularly important, as monitoring programs at large-basin or national scales often require frequent updates over extensive regions. In addition, under conditions involving extremely small water bodies, low land–water contrast, or strong noise, the model can still produce false positives and missed detections, indicating that its robustness on highly complex samples remains limited. Such errors may propagate into systematic biases in estimates of water area, storage, or inundation extent, and thus affect applications such as lake storage assessment, small water body restoration, and urban flood risk analysis [2,9,11,12].

Future work can aim to improve both efficiency and generalization while more closely aligning the model with hydrological and management needs. One direction is to explore lightweight network designs and model compression techniques that reduce computational cost while maintaining segmentation accuracy, enabling large-scale and operational deployment on resource-constrained platforms. Another direction is to leverage multi-source data fusion—for example, jointly using optical and SAR imagery or incorporating DEM and hydrological indicators—to enhance adaptability under varying observation conditions and water regimes. Self-supervised learning and cross-region transfer learning strategies may further strengthen generalization to unseen climatic and ecological zones. Extending HyMambaNet to temporal monitoring and incorporating uncertainty estimation for the segmented water areas would support not only fine-grained static water extraction but also the characterization of water dynamics, drought–flood processes, and long-term ecological restoration outcomes, thereby providing more reliable decision support for water resources management [2,9,11,12].

## 5. Conclusions

This study presents HyMambaNet, a hybrid architecture designed to address the long-standing challenge of balancing fine-grained detail extraction with global context modeling in remote sensing water body segmentation. By integrating the State Space Model (Mamba) into a U-shaped framework, the model achieves linear computational complexity while effectively capturing long-range dependencies—capabilities that conventional CNN-based approaches often struggle to provide.

Extensive experiments on the LoveHY and LoveDA datasets confirm that HyMambaNet consistently outperforms representative CNN-, Transformer-, and hybrid-based segmentation models. The combined use of multi-scale enhancement and frequency-domain feature strengthening significantly improves robustness in challenging scenarios, particularly in extracting small or fragmented water bodies and suppressing interference from shadows, vegetation, and other complex backgrounds. Although the model achieves high precision and recall, its primary advantage lies in maintaining stable performance across diverse hydrological scales and landscape conditions.

From an application perspective, HyMambaNet provides an efficient and scalable solution for high-precision remote sensing analysis, with strong potential in regional water resource monitoring, flood risk assessment, and ecological environmental management. Future work will explore broader cross-regional and cross-sensor validation to further assess the model’s generalization capability, while also improving computational efficiency and integrating uncertainty estimation to support reliable real-time inference on lightweight or edge devices.

## Figures and Tables

**Table 1:** Computational efficiency comparison of Transformer Fusion vs. MambaFusion.

Method	Params (M)	GFLOPS	Inference Time (ms)	Input Size
Transformer Fusion	28.6	64.3	32.7	512 × 512
MambaFusion	17.9	27.4	14.8	512 × 512

**Table 2:** Ablation Study Results on the LoveHY Dataset.

Experiment ID	Module Combination	Precision (%)	Recall (%)	IoU (%)	F1 (%)
A0	UNet	81.18	82.32	67.33	81.75
A1	A0 + VGGBlock	83.45	81.28	68.92	82.35
A2	A1 + MSE	82.91	85.67	70.15	84.26
A3	A2 + SCE	85.13	84.93	71.84	85.02
A4	A3 + WFE	84.76	88.45	73.29	86.58
A5	A4 + MambaFusion	86.21	89.73	74.18	87.94
A6	Full Model	86.88	90.97	74.82	88.87

**Table 3:** Quantitative Comparison of Different Methods on the LoveHY Dataset.

Methods	Precision (%)	Recall (%)	IoU (%)	F1 (%)
Deeplabv3+	84.19	84.55	72.59	84.37
HRNet	80.25	83.28	66.58	81.74
PSPNet	67.79	78.48	58.84	72.77
SegNet	80.05	79.85	66.59	79.95
UNet	81.18	82.32	67.33	81.75
U++	81.22	82.49	68.35	81.85
AttenUNet	81.97	84.08	69.66	83.02
Segformer-B0	70.08	76.61	57.35	73.19
TransUNet	84.14	90.56	71.35	87.26
**Ours**	**86.88**	**90.97**	74.82 *	88.87 *

* indicates that the improvement over the second-best method is statistically significant (*p*-value < 0.05) based on the Wilcoxon signed-rank test. The bold values indicate the best performance in each column.

**Table 4:** Quantitative Comparison of Different Methods on the LoveDA Dataset.

Methods	Precision (%)	Recall (%)	IoU (%)	F1 (%)
Deeplabv3+	86.79	90.21	80.07	88.47
HRNet	86.35	86.07	76.72	86.21
PSPNet	85.31	90.00	78.36	87.61
SegNet	85.64	86.20	75.92	85.92
UNet	86.42	87.33	77.12	86.87
U++	86.56	87.38	77.09	86.97
AttenUNet	86.02	91.51	77.10	88.68
Segformer-B0	81.55	82.59	70.64	82.09
TransUNet	85.99	89.94	78.75	87.91
**Ours**	**88.29**	**91.79**	81.30 *	89.99 *

* indicates that the improvement over the second-best method is statistically significant (*p*-value < 0.05) based on the Wilcoxon signed-rank test. The bold values indicate the best performance in each column.

## Data Availability

Publicly available datasets were analyzed in this study. The LoveDA dataset can be found here: https://github.com/Junjue-Wang/LoveDA (accessed on 21 November 2025). The LoveHY dataset presented in this study is available on request from the corresponding author (mugy390@nenu.edu.cn).
